# Supplementary material for: The pangenome of the human fungal pathogen Cryptococcus neoformans reveals that subtelomeres are hotspots for gene content variation
Source: bioRxiv. 2026 Oct 28:2026.10.27.762246. Preprint. [Version 1] doi: 10.64898/2026.10.27.762246 (PMC13734000; doi:10.64898/2026.10.27.762246)
Supplement: Supplementary file 1 [file NIHPP2026.10.27.762246V1-supplement-1.pdf]

bioRxiv preprint doi: https://doi.org/10.64898/2026.10.27.762246; this version posted October 28, 2026. The copyright holder for this preprint (which was not certified by peer review) is the author/funder, who has granted bioRxiv a license to display the preprint in perpetuity. It is made available under a CC-BY 4.0 International license.

SUPPLEMENTARY FIGURES

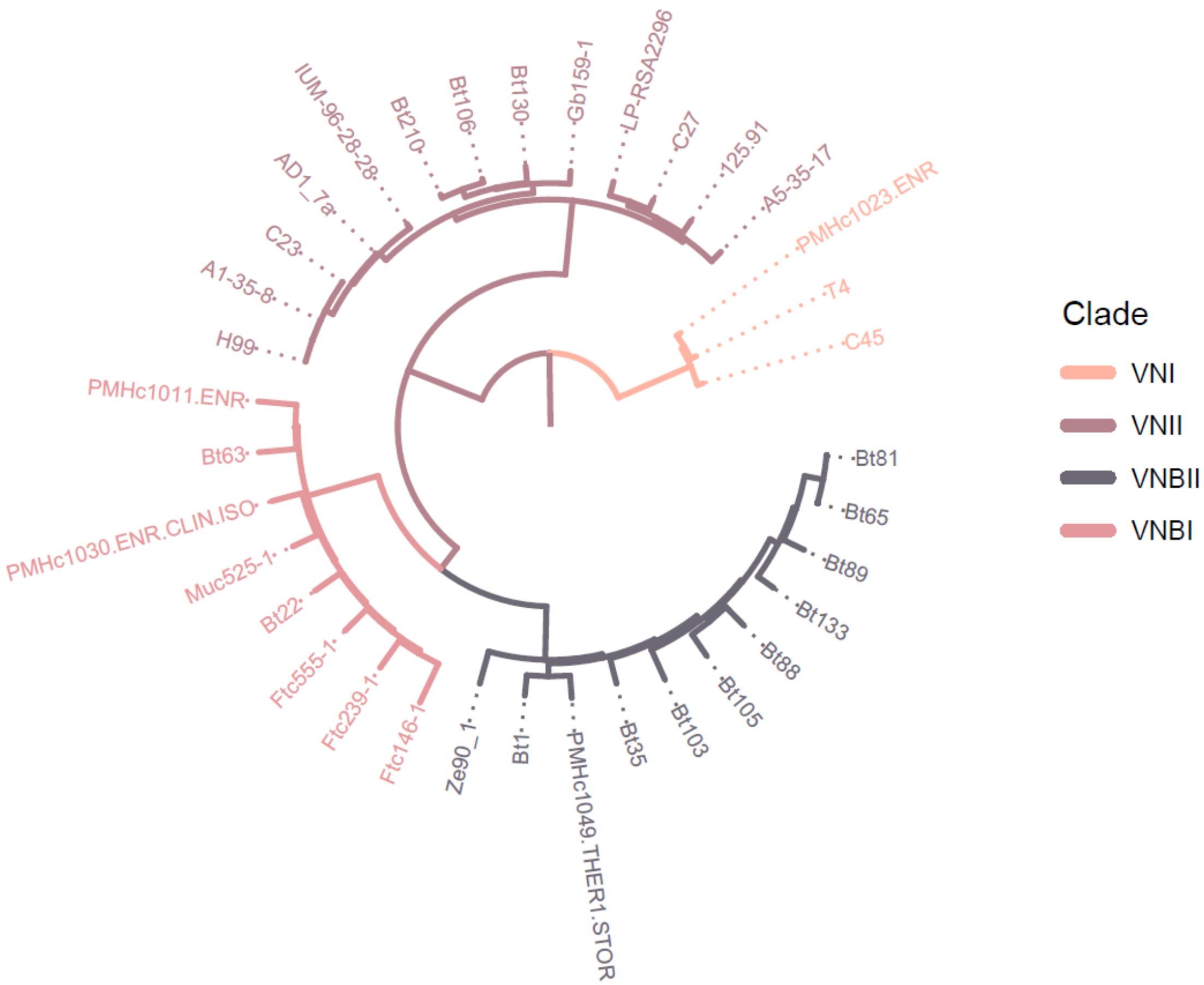

Figure S1. Phylogenetic tree of long-read assemblies included in this study. Tree was calculated by Desjardins et al. 2017 and rooted on VNII.

bioRxiv preprint doi: https://doi.org/10.64898/2026.10.27.762246; this version posted October 28, 2026. The copyright holder for this preprint (which was not certified by peer review) is the author/funder, who has granted bioRxiv a license to display the preprint in perpetuity. It is made available under a CC-BY 4.0 International license.

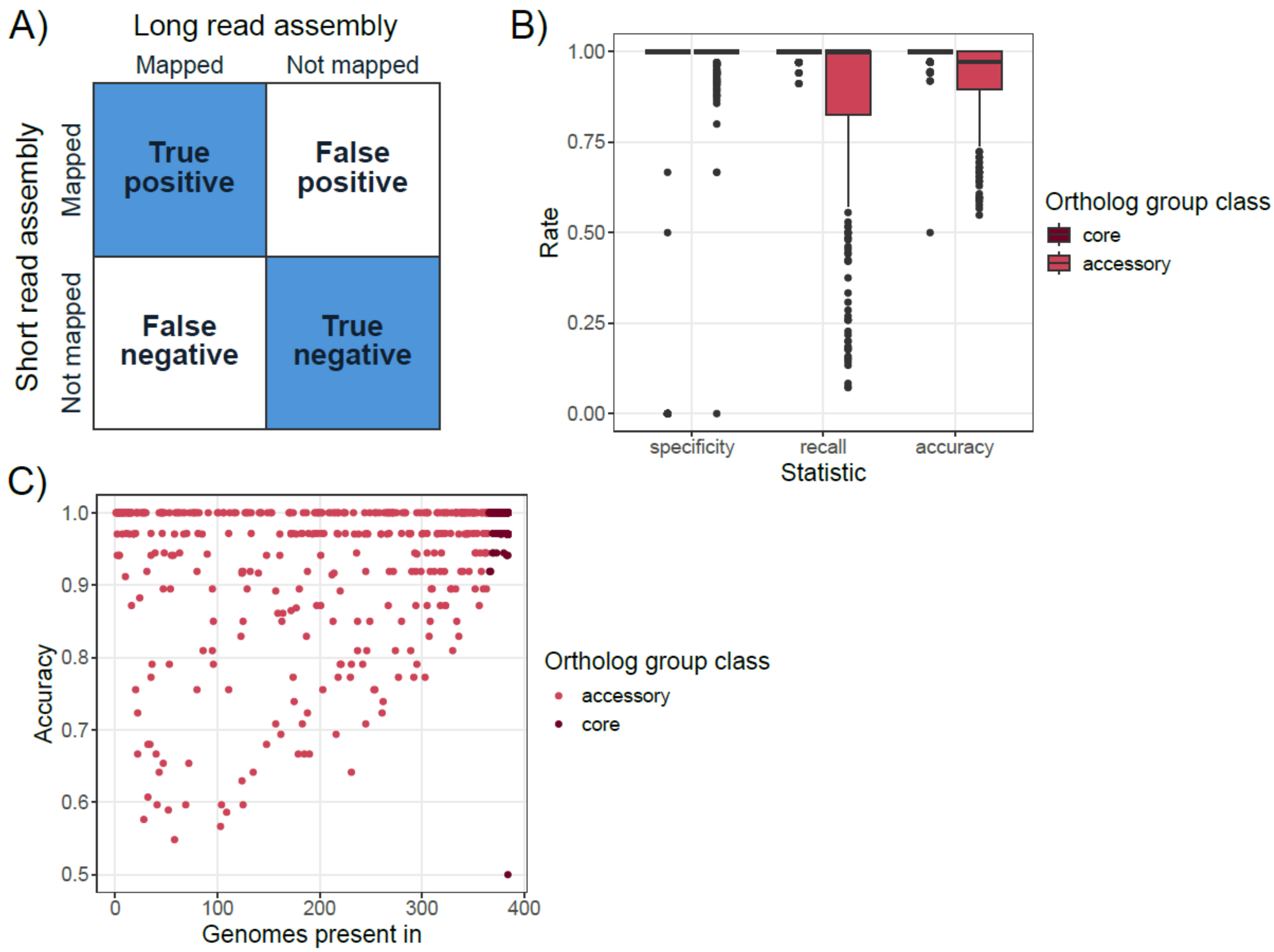

Figure S2. High accuracy of orthologs mapping to long-read assemblies. A) Confusion matrix determining accuracy of short-read assembly ortholog presence/absence compared to long-read assembly mapping data. **B)** Box and whisker plot of summary statistics of core and accessory ortholog groups from mapping short-read annotations to long-read assemblies. Plot shows specificity (1-false positive rate), recall (1- false negative rate), and accuracy for core and accessory ortholog groups. **C)** Ortholog group accuracy compared to the number of genomes that have a given ortholog group.

bioRxiv preprint doi: https://doi.org/10.64898/2026.10.27.762246; this version posted October 28, 2026. The copyright holder for this preprint (which was not certified by peer review) is the author/funder, who has granted bioRxiv a license to display the preprint in perpetuity. It is made available under a CC-BY 4.0 International license.

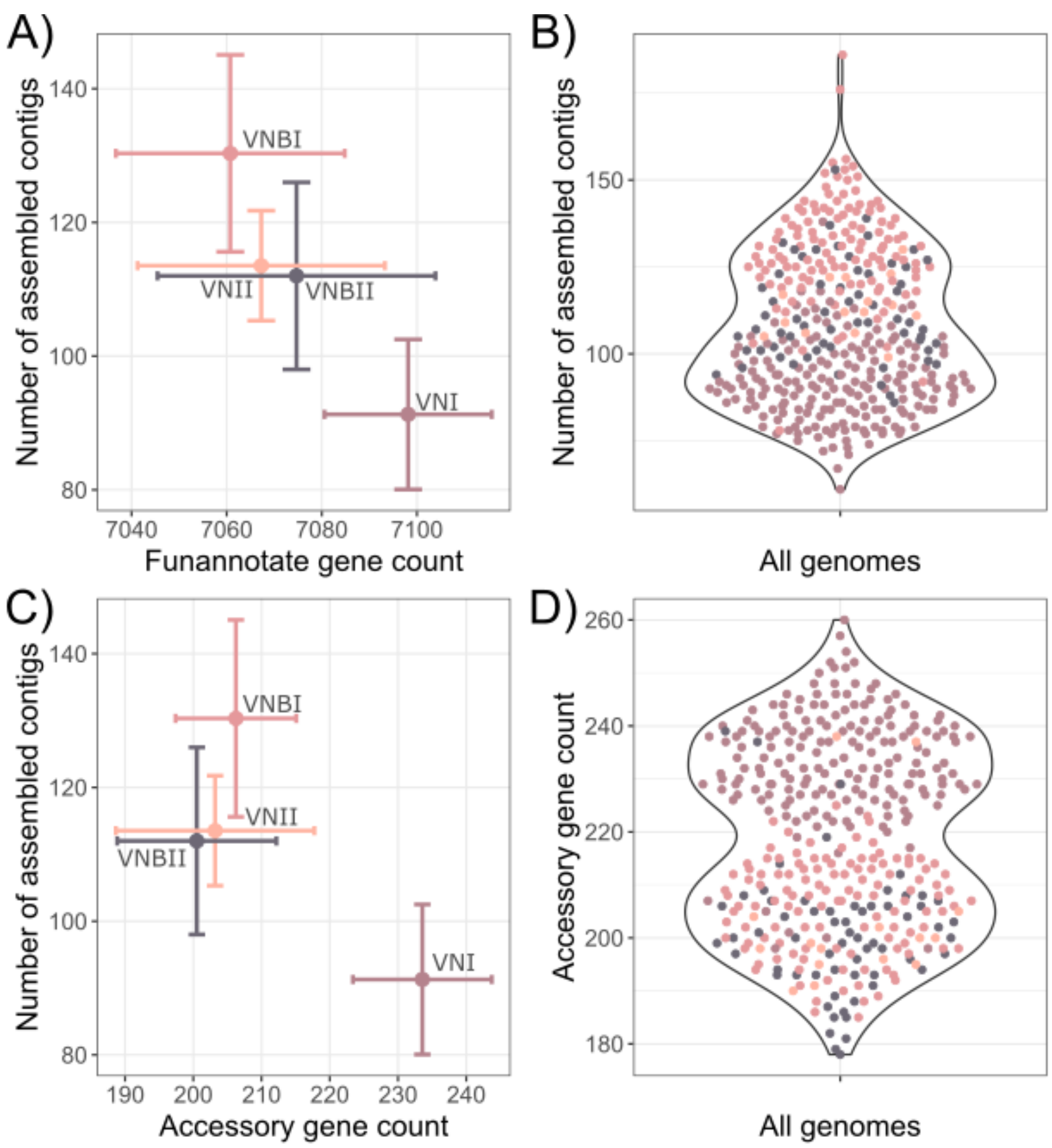

Figure S3. Accessory gene count correlates with assembly quality. A) Direct number of genes called for each molecular type after Funannotate annotation before manual curation. Points show the average for each group and bars are the standard deviation. **B)** Number of assembled contigs per genome. **C)** Final accessory gene count vs. the number of assembled contigs per each molecular type. **D)** Final accessory gene count with points annotated per genome colored by molecular type assignment.

bioRxiv preprint doi: https://doi.org/10.64898/2026.10.27.762246; this version posted October 28, 2026. The copyright holder for this preprint (which was not certified by peer review) is the author/funder, who has granted bioRxiv a license to display the preprint in perpetuity. It is made available under a CC-BY 4.0 International license.

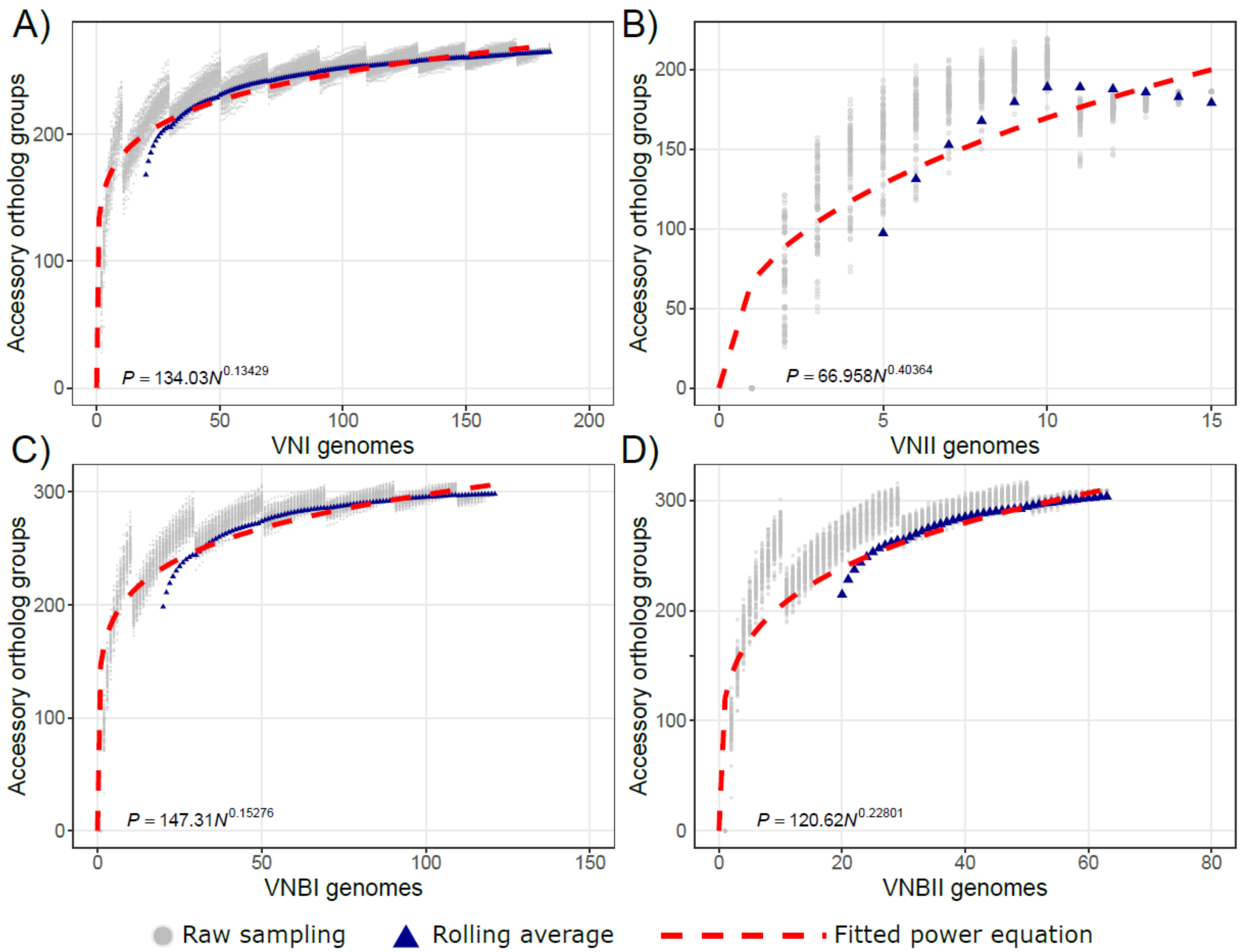

Figure S4. Determining the discovery rate of new genes for each molecular type. 100 randomized samples for the number of new accessory ortholog groups found for each new genome added (grey dots) were taken for each genome within a particular molecular type. Sample sizes were: 184 VNI, 15 VNII, 121 VNBI, and 63 VNBII isolates. Power equations (bottom of graph) were fit (red doted lines) to the rolling average for a given molecular type (blue triangles). **A, C-D)** rolling average was calculated every 20 genomes while **B)** was calculated every 5.
